# Laser cryo-sclerotherapy versus conventional sclerotherapy for reticular veins and telangiectasias: A randomized controlled trial

**DOI:** 10.1016/j.jvsv.2026.102605

**Published:** 2026-09-15

**Authors:** Marcos Maraskin Fonseca, Lara R. Poltronieri, Rodrigo Argenta, Fábio S. Gomes, Lúcia S. Haygert, Glória S. Lazzaretti, Eliane Andrade, Maurício Serra Ribeiro

**Affiliations:** a Postgraduate Program in Surgery, Ribeirão Preto Medical School, University of São Paulo (USP), Ribeirão Preto, São Paulo, Brazil; b Prime Vascular Clinic, Division of Vascular Surgery and Phlebology, Porto Alegre, Rio Grande do Sul, Brazil

**Keywords:** Laser therapy, Varicose veins, Cryo-laser cryo-sclerotherapy, Telangiectasia, Sclerotherapy, Reticular veins

## Abstract

**Objective:**

To evaluate whether the hybrid laser cryo-sclerotherapy (LCS; Nd:Y-AG 1064 nm + 75% hypertonic glucose [HG]) protocol produces superior aesthetic outcomes compared with combined conventional sclerotherapy (CS: 67.5% glucose + 0.3% polidocanol [POL]) for the treatment of lower limb reticular veins and telangiectasias (CEAP C1).

**Methods:**

For this single-center, prospective randomized controlled trial, 60 patients were assessed for eligibility; 4 did not provide informed consent and were not randomized. A total of 56 female patients with CEAP C1 disease and Fitzpatrick phototypes I-III were randomized, with only one lower limb treated per participant: 32 to the LCS group (Nd:YAG 1064 nm + HG75%) and 24 to the CS group (HG67.5% + POL0.3%). Two participants who underwent LCS were subsequently lost to follow-up between Day 1 and Day 30; 54 participants (30 and 24 from the LCS and CS groups, respectively) completed the 90-day follow-up and were analyzed per protocol. Standardized photographs were obtained on Day 1, Day 30, and Day 90, with and without augmented reality (AR; VeinViewer Flex), and were assessed by two blinded external vascular physicians using the Global Aesthetic Improvement Scale (GAIS). Complications, residual pigmentation, and quality of life (Aberdeen Varicose Vein Questionnaire) were evaluated as secondary end points.

**Results:**

Both groups had equivalent baseline characteristics. The LCS group was superior to the CS group in all GAIS outcomes: D1→D90 without AR (*P* = .026; r = 0.34; 95% confidence interval [CI], 0.04-0.58; moderate effect); D1→D90 with AR (*P* < .001; r = 0.53; 95% CI, 0.28-0.72; large effect); D30→D90 without AR (*P* < .001; r = 0.56; 95% CI, 0.31-0.74; large effect); D30→D90 with AR (*P* < .001; r = 0.52; 95% CI, 0.26-0.71; large effect). After Bonferroni correction for these four primary comparisons (α = .0125), the D1→D90 comparison without AR no longer reached significance, whereas the remaining three comparisons did. Temporal analysis revealed a progressive late aesthetic response in the LCS group; 80.0% of participants continued improving between Day 30 and Day 90 vs 29.1% in the CS group (70.8% with No Change). The ecchymosis duration was significantly shorter in the LCS group (median, 5.0 vs 10.0 days; *P* = .012). Severe pigmentation on Day 90 was observed exclusively in the CS group (8.3% vs 0%; *P* = .019; r = 0.22; small effect; power = 36.4%; this finding is exploratory, see the Discussion section). No cases of deep vein thrombosis or ulcer occurred. Aberdeen Varicose Vein Questionnaire score improvement was significant in both groups (*P* < .001), with no intergroup difference. Inter-rater agreement was substantial to near-perfect for GAIS assessments (κ = 0.735-0.913) and perfect for complication grading (κ = 1.000).

**Conclusions:**

The LCS protocol was superior to CS in all GAIS outcomes, with moderate-to-large effect sizes and consistent inter-rater agreement. The aesthetic response was progressive, consolidating between Day 30 and Day 90, with the largest effect size in the late period. AR amplified the magnitude of the detected effects and improved inter-rater agreement, reinforcing its role as a standardizing tool in aesthetic phlebology randomized controlled trials. These statistical findings should be weighed against the higher costs and complexity of LCS relative to CS in a purely cosmetic CEAP C1 population (see the Discussion section).

**Article Highlights**

• **Type of Research:** Single-center, prospective randomized controlled trial
• **Key Findings:** In 54 patients with CEAP C1 disease, laser cryo-sclerotherapy was superior to conventional sclerotherapy across all four Global Aesthetic Improvement Scale comparisons (*P* = .026 to <.001), with a shorter ecchymosis duration (5 vs 10 days; *P* = .012) and less severe residual pigmentation at 90 days (0% vs 8.3%; *P* = .019).
• **Take Home Message:** Laser cryo-sclerotherapy is a safe and effective alternative to conventional sclerotherapy for patients with CEAP C1 disease, despite its higher cost and technical requirements.

Telangiectasias and reticular veins (CEAP C1) are the most prevalent manifestations of vein disease, affecting up to 80% of adult women and representing one of the primary referral diagnoses in vascular surgery and phlebology outpatient clinics.^1^, ^2^, ^3^ Chemical sclerotherapy remains the first-line treatment for these lesions according to vascular society consensus guidelines.^4^ However, recurrence rates are high. Bertanha et al,^5^ in a 36-month follow-up of patients treated with hypertonic glucose (HG) alone or combined with polidocanol (POL), demonstrated significant recurrence over the follow-up period, highlighting the long-term limitations of conventional sclerotherapy (CS) in this population.

Combined protocols, such as HG combined with polidocanol (HG + POL), have produced superior results compared with single agents. Bertanha et al^6^ demonstrated that the combination of POL0.2% + HG70% was significantly more effective than HG75% alone in eliminating reticular veins (95.2% vs 85.4%; *P* < .001). Despite this evidence, the 2021 Cochrane review, which synthesized 35 randomized controlled trials (RCTs) involving >3000 patients, concluded that there was insufficient evidence to establish the superiority of any sclerosing agent over others for long-term outcomes, citing methodological heterogeneity and the lack of standardized response criteria.^4^ In a network meta-analysis, Bontinis et al^7^ refined this conclusion by identifying that the Nd:YAG 1064 nm laser is superior specifically for telangiectasias (β = 1.38; 95% confidence interval [CI], 0.56-2.14), but not for reticular veins; this finding reinforces the hypothesis that combination with a sclerosant is necessary for the complete treatment of both lesion types in the LCS protocol.^8^^,^^9^

The combined use of the Nd:YAG 1064 nm laser with injection sclerotherapy, termed laser cryo-sclerotherapy (LCS) or cryo-laser cryo-sclerotherapy (CLaCS), represents a hybrid approach that exploits the synergy between selective photothermolysis and the chemical action of the sclerosant.^10^ Miyake et al^11^ described the foundations and initial case series of the method, reporting satisfactory vascular clearance in 86% of cases with a low rate of severe complications. The Nd:YAG 1064 nm laser induces immediate but inconsistent changes in reticular veins in the short term, suggesting that the clinically relevant therapeutic response derives from a late vascular remodeling process.^12^^,^^13^ Fonseca et al,^14^ in a triple-blind RCT comparing two sclerosing agents combined with Nd:YAG (HG75% vs POL0.3% + HG67.5%) using the CLaCS protocol, confirmed the efficacy and safety of the hybrid approach, with the superiority of the polidocanol combination in augmented reality (AR) assessment.^15^

To date, no RCT has directly compared a hybrid laser-sclerotherapy strategy with an active combined chemical sclerotherapy protocol, leaving the relative superiority of these approaches undefined. The present study was designed to prospectively evaluate, in a randomized manner and involving blinded external assessors, the LCS protocol (Nd:YAG 1064 nm + HG75%) vs conventional combined sclerotherapy (CS; HG67.5% + POL0.3%) in the treatment of lower limb reticular veins and telangiectasias, with standardized photographic assessment with and without AR.

## Methods

### Study design and population

**Fig 1:**
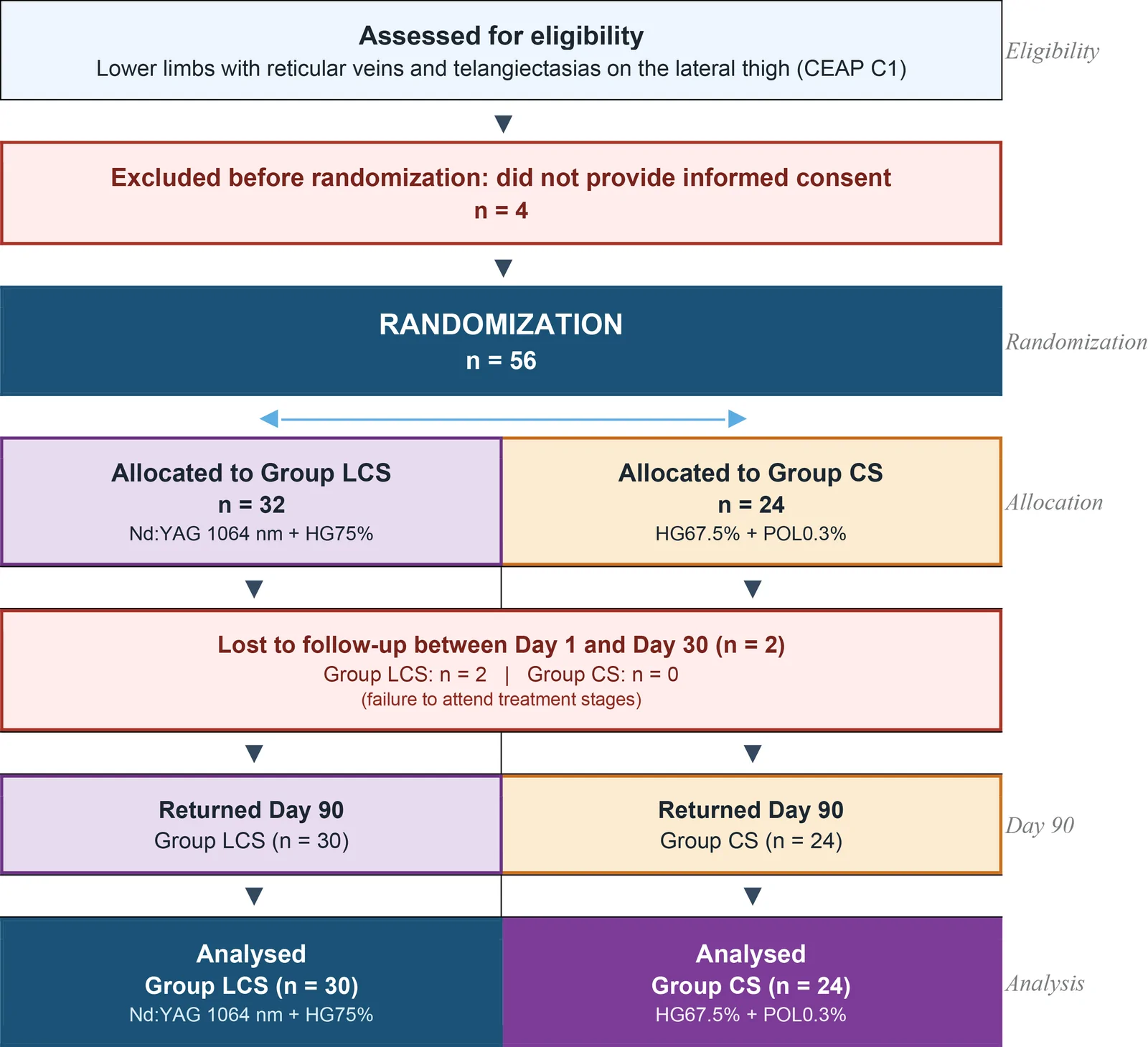
CONSORT flow diagram—Fonseca et al. *CEAP*, Clinical-Etiology-Anatomy-Pathophysiology; *CS*, conventional sclerotherapy (HG67.5% + POL0.3%); *HG*, hypertonic glucose; *LCS*, laser cryo-sclerotherapy (Nd:YAG 1064 nm + HG75%); *POL*, polidocanol.

This prospective, randomized, controlled, single-center clinical trial was conducted at Prime Vascular Clinic (Porto Alegre, RS, Brazil) from November 2024 to June 2025. The study was approved by the Research Ethics Committee of Universidade do Vale dos Sinos (Approval No. 6.072.502; CAAE 68406922.0.0000.5344) and registered in ReBEC (RBR-9wyk6t4). All participants provided written informed consent. This study was conducted in accordance with the Declaration of Helsinki and reported according to the CONSORT 2010 guidelines (Fig 1).

The inclusion criteria were as follows: adult female patients (18-70 years) with telangiectasias and reticular veins on the lateral thigh, CEAP C1, Fitzpatrick phototypes I-III, without reflux in the saphenous veins or deep venous system (confirmed by duplex ultrasound examination), with at least one reticular vein with a minimum length of 5 cm. Eligible patients could not present with a history of allergy to polidocanol or glucose, current or previous deep or superficial vein thrombosis, known thrombophilias, anticoagulant use, dermatitis at the treatment site, restricted mobility, or uncontrolled systemic diseases. Only one lower limb per participant was included in the study. When both limbs fulfilled the eligibility criteria, the limb selected for treatment was chosen by the participant before randomization according to their personal preference, avoiding within-patient clustering and ensuring the statistical independence of observations.

The exclusion criteria were as follows: failure to attend treatment stages after randomization.

### Sample size calculation

The sample size was calculated for the comparison of two independent means (primary outcome: Global Aesthetic Improvement Scale [GAIS] score), considering two-tailed α = .05, power = 80%, Cohen's d = 0.80, minimum clinically relevant difference = 1 point on GAIS, and estimated standard deviation = 1.2 (based on Fonseca et al). The calculation yielded a minimum of 23 participants per group, with a 20% attrition margin and with a total of 56 participants. Sixty patients were assessed for eligibility (Fig 1); four did not provide informed consent and were not randomized. Fifty-six patients were randomized (32 to Group 1 and 24 to Group 2); 54 completed the 90-day follow-up (30 in Group 1 and 24 in Group 2).

### Randomization and blinding

Before the enrollment of any participant, allocation was performed by simple randomization using a sequence of 60 random numbers generated electronically (stattrek.com, November 9, 2024), ensuring unpredictability and traceability. The full sequence (corresponding to allocations “01” and “02”) was generated in a single step before enrollment began; each eligible patient received a consecutive number from 01 to 60 in order of inclusion, and allocation was conducted following that pregenerated sequence exactly, without investigator discretion at the point of enrollment. Each participant received a blinded numerical identifier used in all records. Two external assessors remained blinded to group allocation throughout photographic analysis, which was conducted using a structured evaluation table with criteria adapted for reticular veins and telangiectasias (based on the classic GAIS anchors). The principal operator was aware of allocation due to technical necessity (open-label for the operator), a recognized inherent limitation of interventional RCTs with different protocols.

### Interventions

Group 1—LCS: long-pulse transdermal laser (Etherea MX, Vydence) with fixed parameters: spot size of 6 mm, fluence of 70 J/cm^2^, pulse duration of 15 ms, and maximum of 50 shots, with 10% spot size overlap and two passes per target vein. Immediately afterward, 75% HG (HealthTeck) was injected using the CS technique: a maximum of 40 punctures with a 27G½ needle, maximum total volume of 3 mL (≤1 mL per reticular vein; ≤0.2 mL per telangiectasia).

Group 2—CS: CS using a solution prepared by diluting 3% polidocanol in HG (final concentration: HG67.5% + POL0.3%), with the same puncture and volume parameters described for Group 1. Skin cooling with the Siberian-FIT Vydence device was applied during the procedure in both the LCS and CS groups.

The treatment zone was defined as a 10 × 8-cm rectangle (80 cm^2^) on the lateral thigh. All procedures were performed by a single operator certified in vascular surgery in a single session per limb.

### Outcome assessment

**Fig 2:**
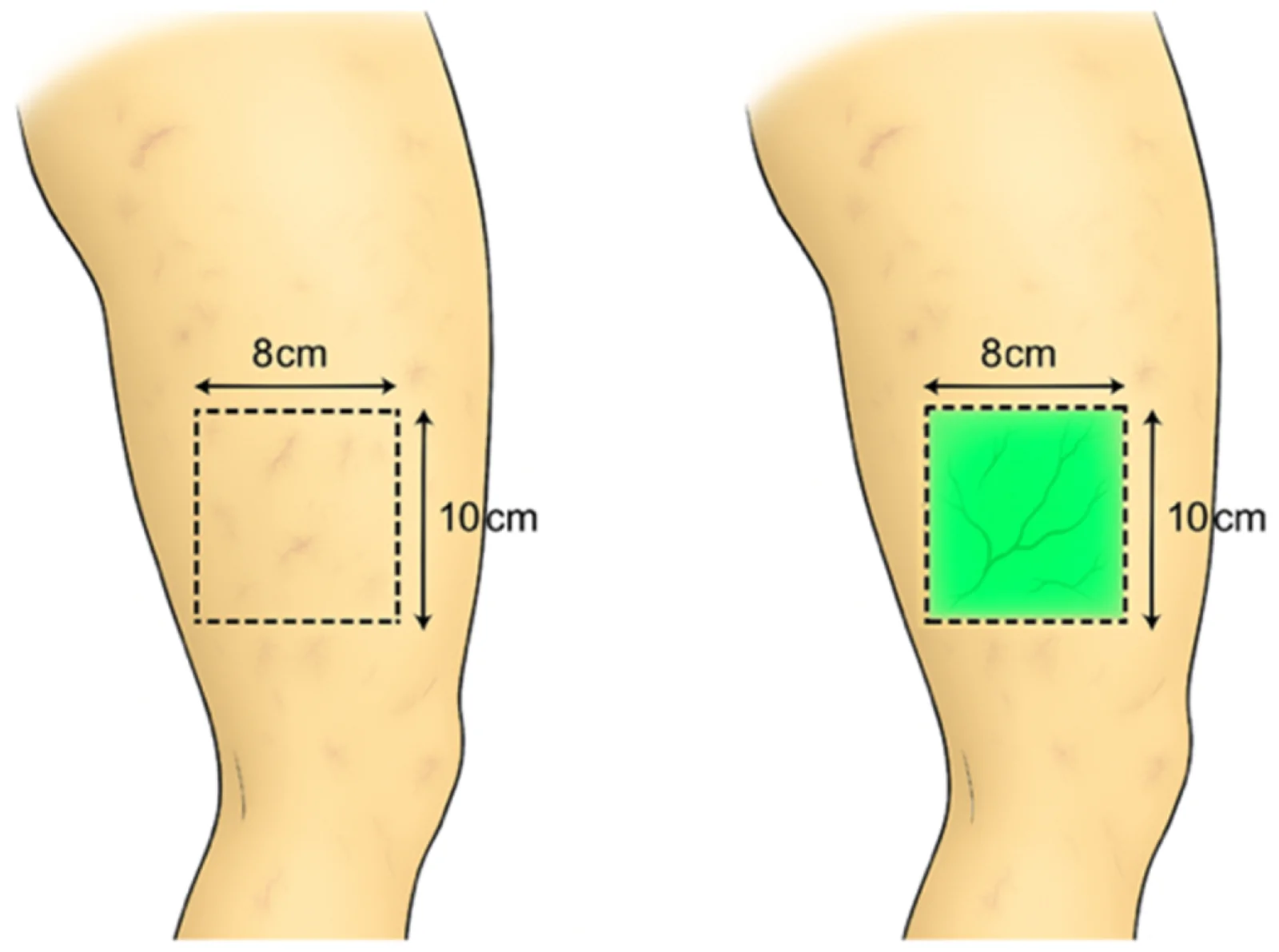
Schematic model of study demarcation and photography. Images without and with augmented reality using the VeinViewer Flex device.

Standardized photographs were obtained on Day 1 (pre-procedure), Day 30, and Day 90 using a Canon EOS Rebel SL2 camera (24.2 MP), with a focal distance of 30 cm, controlled lighting, patient in orthostatism, with a white dermographic template. Each photographic session generated two records: one without a visualization device and one with the VeinViewer Flex device (near-infrared AR technology, NIR of 760 nm, projecting the subcutaneous venous network directly onto the skin in bright green, revealing the vessels not detectable by the naked eye) (Fig 2).

The two blinded external vascular physicians independently assessed paired photographs (pre- and post-treatment) using GAIS, classifying outcomes into five ordinal categories: Worse (1), No Change (2), Improved (3), Much Improved (4), and Excellent (5), following the ascending “worse-to-very-much-improved” anchor ordering originally described by Narins et al,^16^ which is the ordering conventionally used across the aesthetic outcomes literature. The outcomes analyzed were GAIS D1→D90 and GAIS D30→D90, with and without AR.

The secondary outcomes were the occurrence and duration of ecchymosis, pain, phlebitis, matting, hyperpigmentation, deep vein thrombosis, and ulceration (assessed on Day 30); residual pigmentation on Day 90 (ordinal scale: none, small, and important); quality of life using the Aberdeen Varicose Vein Questionnaire (modified AVVQ) on Day 1 and Day 90.

### Statistical analysis

Continuous variables are described as mean ± standard deviation or median (interquartile range [IQR]) according to the distribution assessed using the Shapiro-Wilk test. Between-group comparisons were performed using Student's *t*-test (normal variables) or the Mann-Whitney *U* test—equivalently and the Wilcoxon rank-sum test—for non-normally distributed or ordinal variables. This term is used consistently for all independent-group comparisons throughout the tables. Categorical variables were compared using the *χ*^2^ test or Fisher exact test. The effect size for between-group comparisons was estimated using the rank-biserial correlation coefficient (r), with 95% CIs calculated by bootstrap (2000 resamples, percentile method). The paired Wilcoxon test was used for within-group and before-after comparisons over follow-up. For the four primary GAIS comparisons, a Bonferroni-corrected significance threshold (α = .0125) was applied post hoc to account for multiple testing, and the results are reported against both the nominal and the corrected threshold. Inter-rater agreement was measured using the linear weighted Cohen's Kappa, with bootstrap 95% CI. The significance level was set at α = .05 (nominal, before correction). An intention-to-treat sensitivity analysis was additionally performed for the two participants lost to follow-up after randomization (both in the LCS group, between Day 1 and Day 30; Fig 1), who were retained in the LCS group and assigned the least favorable (Worse) and most favorable (Excellent) GAIS category in the worst-case and best-case scenarios, respectively, with the primary Mann-Whitney comparisons re-run under both assumptions (LCS n = 32, CS n = 24; the CS group required no imputation, as it had no post-randomization losses). Analyses were conducted in R (version 4.x) with R Commander interface, and study power was determined using G∗Power.

## Results

### Participant flow and baseline characteristics

Sixty patients were assessed for eligibility, all of whom met the clinical inclusion criteria. Four did not provide informed consent and were not randomized; thus, 56 of 60 patients assessed were enrolled and randomized (Fig 1)—32 to the LCS group and 24 to the CS group. Two participants who underwent LCS were subsequently lost to follow-up between Day 1 and Day 30 (failure to attend treatment stages); no losses occurred in the CS group. Fifty-four participants completed the 90-day follow-up: 30 in the LCS group and 24 in the CS group. Both groups had similar baseline characteristics (Table I), including the Fitzpatrick phototype distribution (predominantly types II-III) and baseline AVVQ scores (*P* = .790 and *P* = .619, respectively).

**Table I:** Baseline characteristics of the study population

Variable	Group 1—LCS (n = 30)	Group 2—CS (n = 24)
Age, years, mean ± SD	47.97 ± 11.81	46.79 ± 12.51
BMI, kg/m^2^, mean ± SD	26.69 ± 5.74	28.10 ± 4.16
Regular physical activity, n (%)	23 (76.7%)	16 (66.7)
Active smoking, n (%)	1 (3.3)	1 (4.2)
Systemic arterial hypertension, n (%)	4 (13.3)	5 (20.8)
Diabetes mellitus, n (%)	1 (3.3)	2 (8.3)
Family history of varicose veins, n (%)	21 (70.0)	22 (91.7)
Oral contraceptive use, n (%)	9 (30.0)	7 (29.2)
Previous pregnancies, n (%)	16 (53.3)	9 (37.5)
Fitzpatrick phototype II, n (%)	17 (56.7)	12 (50.0)
Fitzpatrick phototype III, n (%)	13 (43.3)	11 (45.8)
Baseline AVVQ (median, IQR)	19.4 (11.1-30.6)	21.6 (16.0-27.8)

*AVVQ*, Aberdeen Varicose Vein Questionnaire; *BMI*, body mass index; *IQR*, interquartile range; *SD*, standard deviation.

Student *t*-test^1^ or Mann-Whitney *U*^2^; *χ*^2^ or Fisher exact test^4^ as applicable.

### Procedural data

The maximum reticular vein diameter on B-mode ultrasound images was similar between the groups (1.40 vs 1.45 mm; *P* = .944), confirming anatomical homogeneity. The number of punctures did not differ (*P* = .235). The total injected volume was significantly lower in the LCS group (1.70 vs 2.20 mL; *P* = .007), suggesting that laser pretreatment reduces sclerosant requirements (Table II).

**Table II:** Procedural parameters (Day 1)

Variable	Group 1—LCS (n = 30)	Group 2—CS (n = 24)	*P* value
No. laser shots, median (IQR)	44.5 (39.0-48.75)	0 (0-0)	<.001
No. punctures, median (IQR)	17 (13.25-21)	14 (12.75-17.25)	.235
Max. reticular vein diameter on B-mode (mm), median (IQR)	1.40 (1.30-1.78)	1.45 (1.30-1.63)	.944
Total injected volume (mL), median (IQR)	1.70 (1.50-2.10)	2.20 (1.88-2.50)	.007^a^

*CS*, Conventional sclerotherapy; *IQR*, interquartile range; *LCS*, laser cryo-sclerotherapy.

a *P* = .007 (Mann-Whitney *U*).

### Clinical complications at 30 days

No cases of deep vein thrombosis or cutaneous ulceration were recorded in either group. Ecchymosis was frequent in both groups (LCS: 90.0%; CS: 100.0%; *P* = .245), but its duration was significantly shorter in the LCS group (5.0 vs 10.0 days; *P* = .012). Phlebitis (0% vs 8.3%; *P* = .193) and matting (0% vs 8.3%; *P* = .193) were numerically more common in the CS group, without reaching statistical significance. The details are presented in Table III.

**Table III:** Clinical complications at 30 days

Variable	Group 1—LCS (n = 30)	Group 2—CS (n = 24)	*P* value
Pain, n (%)	12 (40.0)	12 (50.0)	.584
Ecchymosis, n (%)	27 (90.0)	24 (100)	.245
Ecchymosis duration, days, median (IQR)	5.0 (3.0-10.0)	10.0 (6.8-13.5)	.012^a^
Phlebitis, n (%)	0 (0)	2 (8.3)	.193
Matting, n (%)	0 (0)	2 (8.3)	.193
DVT, n (%)	0 (0)	0 (0)	1.000
Ulcer, n (%)	0 (0)	0 (0)	1.000
Residual pigmentation on Day 90			
No pigmentation, n (%)	25 (83.3)	19 (79.2)	–
Small pigmentation, n (%)	5 (16.7)	3 (12.5)	–
Significant pigmentation, n (%)	0 (0)	2 (8.3)	–
Mann-Whitney *U* test	–	–	.019
r = 0.22 (95% CI: 0.09-0.49); power 36.4%	–	–	small effect

*CS*, Conventional sclerotherapy; *CI*, confidence interval; *DVT*, Deep vein thrombosis; *IQR*, interquartile range; *LCS*, laser cryo-sclerotherapy.

*χ*^2^ or Fisher exact test as applicable.

a *P* < .05 (Mann-Whitney *U*).

### Quality of life (AVVQ) and patient perception

AVVQ scores improved significantly in both groups between Day 1 and Day 90 (*P* < .001 in each group). The median absolute change was 4.35 points in the LCS group and 2.45 points in the CS group, with no statistically significant intergroup difference (*P* = .937). The post hoc power for this comparison was 9.1%, indicating that the study was not powered to detect small functional differences in patients with CEAP C1 disease.

Regarding the subjective perception of aesthetic outcomes assessed by patients on Day 90, the LCS group had a significantly higher proportion of patient-reported Very Good/Excellent ratings compared with the CS group (70.0% vs 20.9%; *P* < .001). No patient in the LCS group rated the result as unsatisfactory, whereas the CS group had a higher frequency of neutral or negative ratings. These findings reinforce the concordance between objective photographic assessment by blinded evaluators and the clinical perception reported by patients. The details are presented in Table IV.

**Table IV:** Patient subjective perception of aesthetic outcomes on Day 90 by treatment group

Subjective perception of outcome	Group 1—LCS (n = 30)		Group 2—CS (n = 24)		*P* value
Category	n	%	n	%	
Very Good/Excellent	21	70.0	5	20.9	<.001^a^
Good	9	30.0	11	45.8	
Regular/Poor	0	0.0	8	33.4	
Total	30	100	24	100	

*CS*, Conventional sclerotherapy (HG67.5% + POL0.3%); *LCS*, laser cryo-sclerotherapy (Nd:YAG 1064 nm + HG75%).

Patient self-report aesthetic satisfaction: Very Good/Excellent = satisfactory outcome; Good = partially satisfactory outcome; Regular/Poor = unsatisfactory outcome.

a Mann-Whitney test *U* (ordinal analysis); *P* < .001.

### Global Aesthetic Improvement Scale

**Fig 3:**
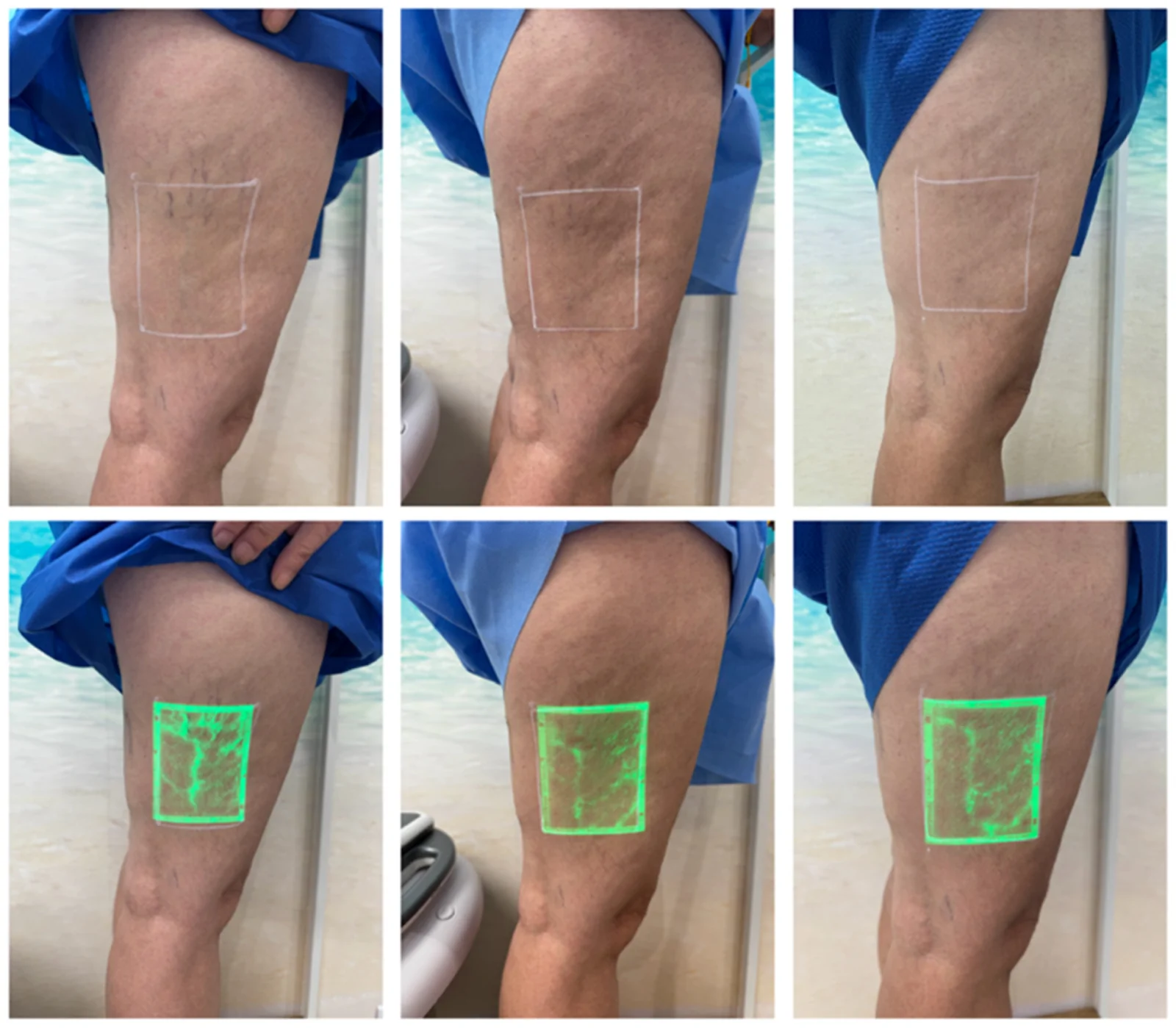
Treatment follow-up photographs on Day 1, Day 30, and Day 90 with and without augmented reality (VeinViewer Flex). Improvement of the telangiectasia network from Day 1 to Day 30 and from Day 30 to Day 90.

**Fig 4:**
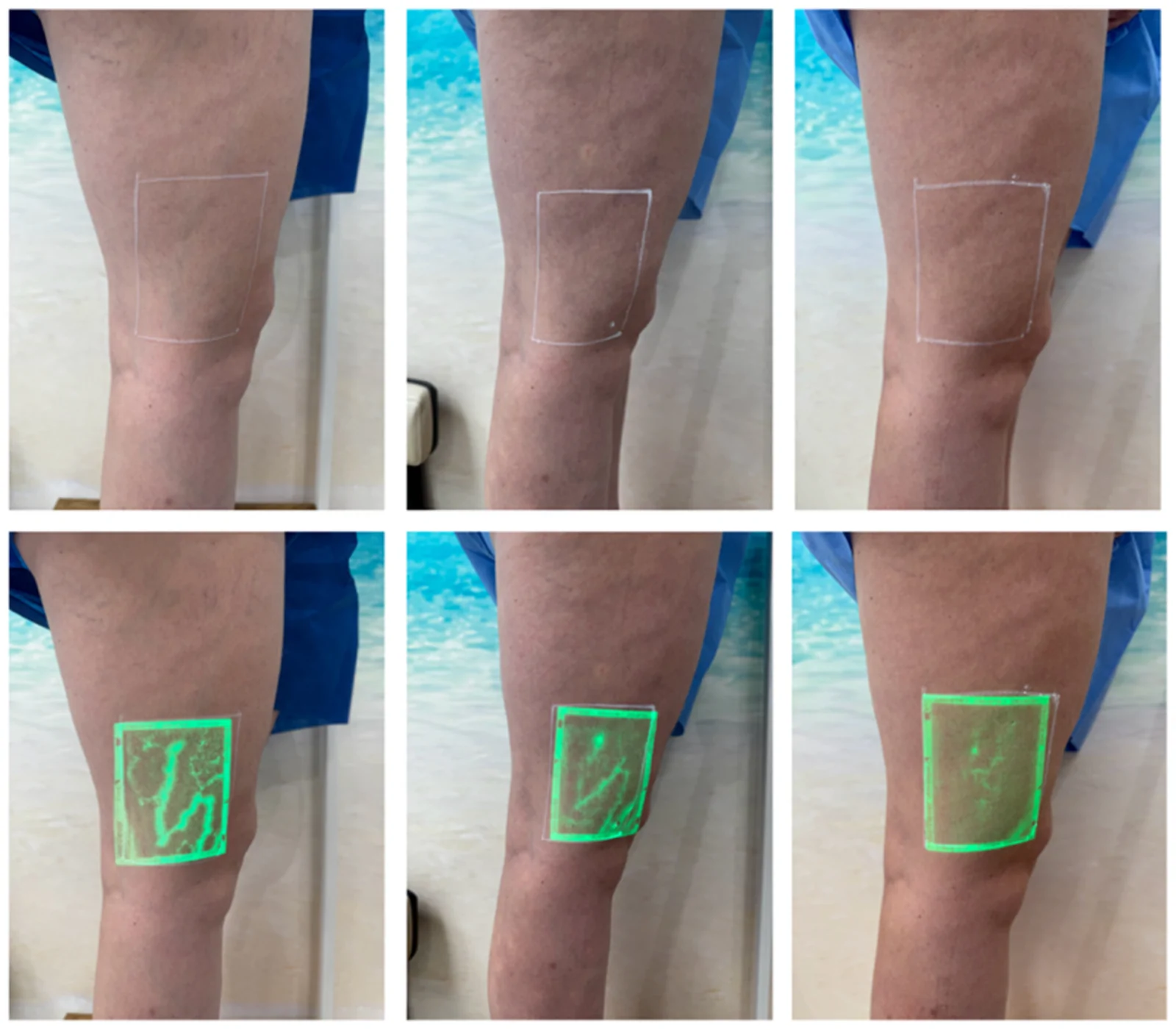
Treatment follow-up photographs on Day 1, Day 30, and Day 90 with and without augmented reality (VeinViewer Flex). Additional vascular clearance from Day 30 to Day 90 in Augmented reality (AR).

The GAIS results are summarized in Table V. The LCS group was superior to the CS group in all four comparisons. Without AR, 73.4% of participants who underwent LCS were rated as Much Improved or Excellent at D1→D90 vs 37.5% in the CS group (*P* = .026; Fig 3); no participant who underwent LCS was rated as No Change or Worse. With AR in the same period, favorable outcomes reached 86.7% vs 37.5% (*P* < .001). In the D30→D90 period, 80.0% of participants who underwent LCS showed further improvement after Day 30 compared with 29.1% in the CS group, where 70.8% showed no additional change (*P* < .001). With AR in the late period, 70.0% and 25.0% of participants in the LCS and CS groups, respectively, had favorable outcomes (*P* < .001); worsening was recorded exclusively in the CS group (8.3%; Fig 4). After the application of the prespecified Bonferroni correction (α = .0125) for these four comparisons, the D1→D90 comparison without AR (nominal *P* = .026) did not retain significance, whereas the remaining three comparisons (all nominal *P* < .001) remained significant. We present this correction to be conservative while noting that the uncorrected result is also consistent in direction and magnitude with the corrected ones. All statistical details are presented in Table V.

**Table V:** Global Aesthetic Improvement Scale results by blinded assessors

Outcome	G1 Med (IQR)	G2 Med (IQR)	W	*P* value	R	95% CI	Magnitude (study power)
D1→D90 without AR	4 (3-5)	3 (3-4)	482	.026^a^	0.34	0.04-0.58	Moderate (74.4%)
D1→D90 with AR	4 (4-5)	3 (3-4)	552.5	<.001^a^	0.53	0.28-0.72	Large (99.4%)
D30→D90 without AR	3 (3-4)	2 (2-2)	563	<.001^a^	0.56	0.31-0.74	Large (99.8%)
D30→D90 with AR	3 (2-4)	2 (2-2)	548.5	<.001^a^	0.52	0.26-0.71	Large (99.2%)

*AR*, Augmented reality; *IQR*, interquartile range; *Med*, median; *r*, rank-biserial correlation (effect size) with 95% confidence interval (*CI*) by bootstrap (2000 resamples); *W*, Wilcoxon statistic.

Effect magnitude: 0.10-0.29, small; 0.30-0.49, moderate; ≥0.50, large.

Scale: 1 = Worse, 2 = No Change, 3 = Improved, 4 = Much Improved, 5 = Excellent.

a *P* < .05 two-tailed.

The temporal analysis of GAIS scores revealed a pattern of progressive late vascular clearance in the LCS group. Although 70.8% of CS participants showed No Change in the D30→D90 interval (without AR), the LCS group had 80.0% favorable outcomes in the same period, a pattern consistent across both blinded evaluators and both photographic modalities. Notably, worsening in the late period was recorded exclusively in the CS group (8.3% with AR).

**Fig 5:**
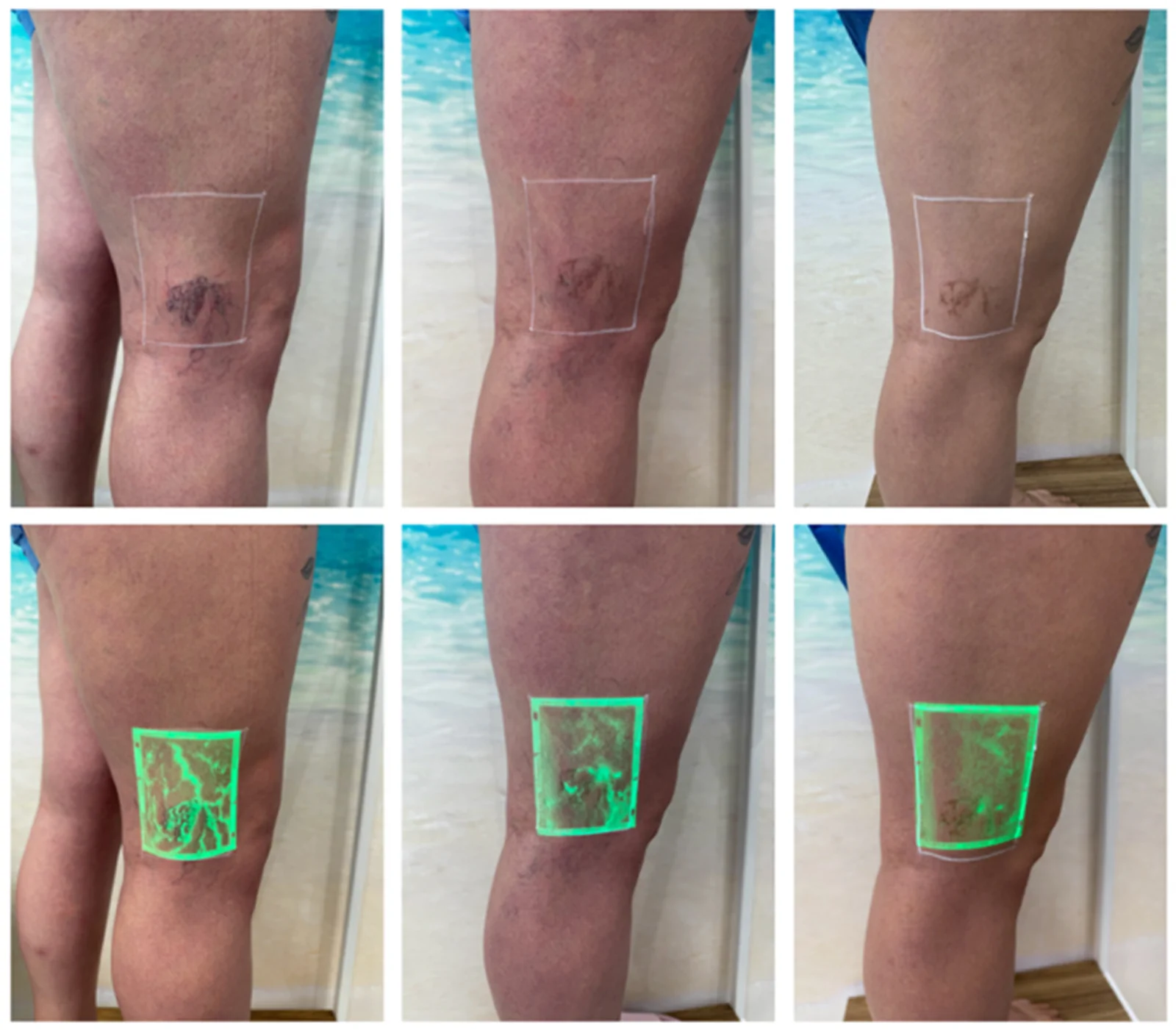
Significant residual pigmentation—CS group. *CS*, Conventional sclerotherapy.

**Fig 6:**
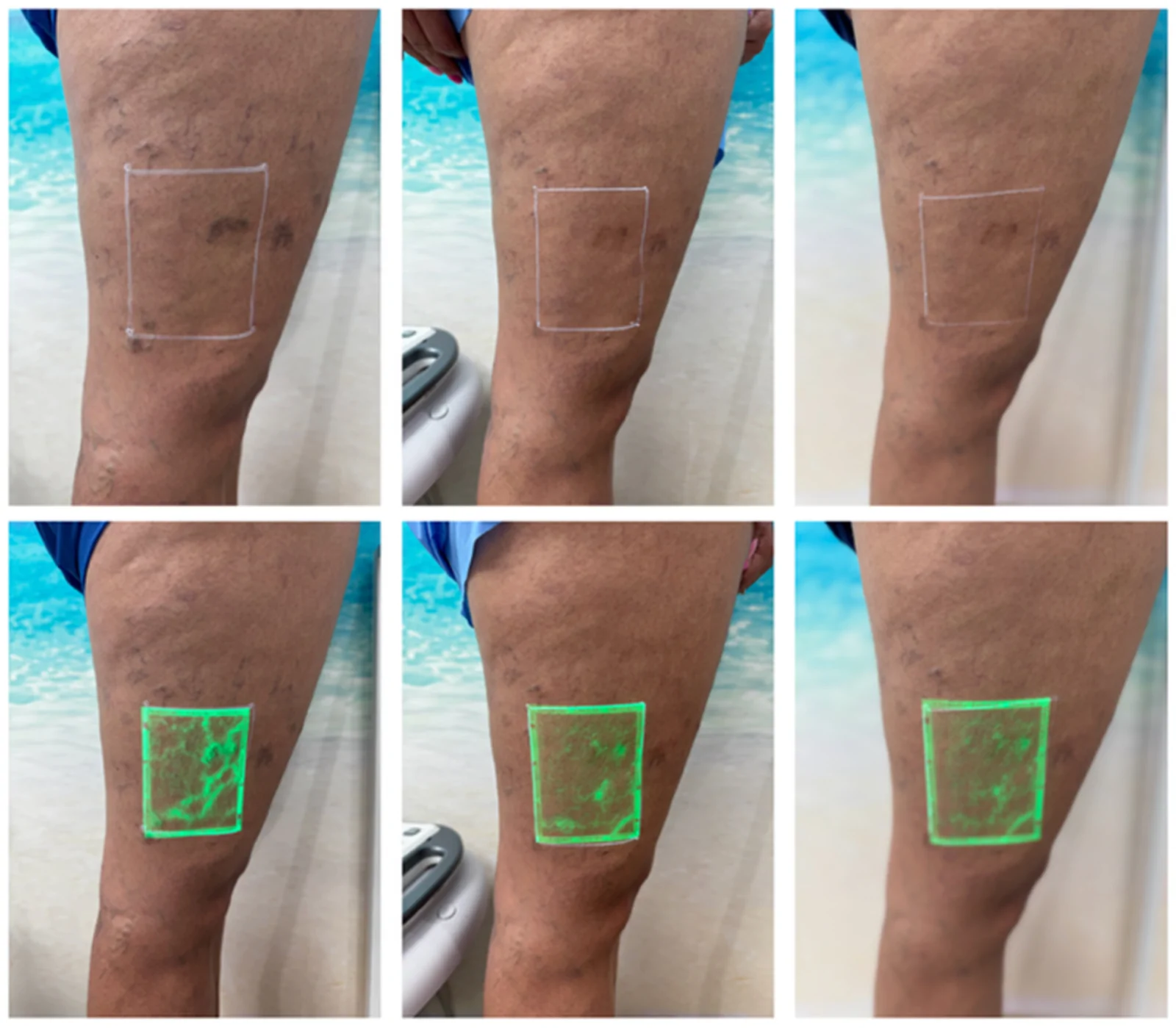
Small residual pigmentation—LCS group. *LCS*, Laser cryo-sclerotherapy.

Residual pigmentation on Day 90 differed significantly between the groups (*P* = .019). Severe pigmentation was found only in the CS group (8.3%; Fig 5), and all participants who underwent LCS had absent or mild pigmentation (Fig 6). The effect size was small (r = 0.22) with low post hoc power (36.4%), indicating that this finding should be considered an exploratory secondary finding. The distribution by grade is shown in Table III. No severe complications were recorded in either group (*P* = 1.000).

### Inter-rater agreement

Inter-rater agreement was substantial to near-perfect for all GAIS assessments (κ = 0.735-0.913) and perfect for complication grading (κ = 1.000). Pigmentation grading also showed perfect agreement (κ = 1.000), with intergroup analysis performed separately using the Mann-Whitney *U* test (Table VI). The lowest coefficient was observed for GAIS D1→D90 without AR (κ = 0.735), consistent with the greater difficulty in discriminating adjacent categories (Improved/Much Improved) without technological assistance, whereas the highest was found for GAIS D1→D90 with AR (κ = 0.913), confirming that AR standardizes assessment and reduces interobserver variability (Table VI).

**Table VI:** Inter-rater agreement—Weighted linear Cohen Kappa (95% CI by bootstrap)

Outcome	A2 × A3 (κ)	Classification
GAIS D1→D90 without AR	0.735	Substantial
GAIS D30→D90 without AR	0.843	Near-perfect
GAIS D1→D90 with AR	0.913	Near-perfect
GAIS D30→D90 with AR	0.843	Near-perfect
Pigmentation D90	1.000	Perfect
Complications	1.000	Perfect

*AR*, Augmented reality; *A2, A3*, assessors 2 and 3; *CI*, confidence interval.

Classification per Landis and Koch: 0.61-0.80, Substantial; 0.81-1.00, Near-perfect/Perfect.

Kappa calculated with bootstrap 95% CI (2000 resamples).

## Discussion

Our results demonstrated that the LCS group was superior to the CS group in all four GAIS comparisons, with moderate-to-large effect sizes (r = 0.34-0.56) and consistent inter-rater agreement across all primary outcomes. Three of the four comparisons also remained significant after conservative Bonferroni correction for multiple testing (see the Methods and Results sections). Both the blinded evaluators agreed consistently, reinforcing the robustness of these findings.

AR played a relevant methodological role. In the D1→D90 period, both evaluators detected between-group differences with and without AR, but with a consistently larger effect size with AR (r = 0.53 vs 0.34). Inter-rater agreement was also higher with AR (κ = 0.913 vs 0.735 for D1→D90).^17^ We believe that AR reduces observer subjectivity by making subtle vascular clearance more easily identifiable.

From the pathophysiological perspective, the superiority of the LCS group in terms of late outcomes supports the hypothesis of photothermal-osmotic synergy. The Nd:YAG laser promotes focal endothelial injury and local blood flow reduction through microthrombosis, increasing the effective concentration and contact time of the subsequent sclerosant with the damaged venous wall.^11^ Histological studies confirm the occurrence of subendothelial blistering, tunica media edema, elastic fiber destruction, and transient vascular spasm following transdermal Nd:YAGexposure, creating a favorable microenvironment for the osmotic action of HG.^12^ The immediate laser-induced vasospasm may also contribute to endothelial dysfunction and transient luminal narrowing, reducing the vessel caliber and enhancing sclerosant contact with the vessel wall, which may thus promote endothelial injury. This transient reduction in the luminal diameter may also partially explain the lower sclerosant volume required in Group 1, as a smaller intravascular volume would theoretically require less HG to achieve effective endothelial exposure and vascular obliteration.^13^

Three mechanisms may explain this pattern. First, laser-induced mural fibrosis takes 4 to 12 weeks to occur, meaning that the aesthetic gain continues to accumulate after Day 30.^12^^,^^13^ Second, the LCS group required a lower sclerosant volume (1.70 vs 2.20 mL; *P* = .007), suggesting that the laser enhanced the chemical effect and produced more focal obliteration with less sclerosant dispersal. Third, the earlier resolution of the inflammatory phase in the LCS group, reflected by the shorter ecchymosis duration (5.0 vs 10.0 days; *P* = .012), implies that Day 30 photographs in the CS group may still capture residual edema and hemosiderin, artificially masking the result at this time point.

The absence of deep vein thrombosis or cutaneous ulceration in either group (*P* = 1.000) is consistent with the venous-capillary reflux mechanism proposed by Miyake et al,^18^ who demonstrated in an in vitro and experimental rabbit model that both the sclerosant viscosity and vessel caliber determine the risk of ischemic skin necrosis after sclerotherapy.

Moreno-Moraga et al^19^ demonstrated the superiority of the Nd:YAG 1064 nm laser combined with polidocanol microfoam over sclerotherapy alone in an RCT of 517 limbs with a 3-year follow-up. Miyake et al^20^^,^^21^ documented aesthetic stability at 11 years in a patient treated with AR-guided CLaCS. Our study adds controlled evidence of the temporal response pattern between Day 30 and Day 90, which cannot be captured by studies with a single end point.

Nasser et al^15^ compared CLaCS (70% dextrose) vs polidocanol sclerotherapy in 392 patients and found superior clearance rates with CLaCS (100% vs 85.3%; *P* < .001) and lower hyperpigmentation. Our study differs in that it used a single session, serial photographic assessment with and without AR and the AVVQ as a functional outcome, enabling the characterization not only of the magnitude but also the temporal pattern of the aesthetic response.

In a systematic review, Baldaia et al^22^ confirmed that the long-pulsed Nd 1064 nm laser is effective for lower limb veins up to 3 mm, with greater efficacy in vessels <1 mm. Similarly, in a network meta-analysis including 19 studies involving 1356 patients, Bontinis et al^7^ demonstrated the superiority of Nd 1064 nm over sclerosants for telangiectasias, and HG75% also ranked among the highest-performing agents. More recently, Sathler-Melo and Faccini^23^ reported a retrospective cohort treated with a hybrid laser-cryo-sclerotherapy protocol for CEAP C1 and C2 (low) disease, including reticular veins and small varicose veins up to approximately 3 mm in diameter, with favorable safety and aesthetic outcomes. Although it was retrospective, uncontrolled, and therefore not directly comparable to our randomized design, this report is consistent with our findings and suggests the LCS approach may be extendable beyond purely telangiectatic C1 disease, a hypothesis that requires prospective confirmation. To the best of our knowledge, our study is the first RCT to compare a hybrid laser-sclerotherapy protocol (LCS; Nd + HG75%) with an active combined chemical sclerotherapy strategy (CS; HG67.5% + POL0.3%) using serial photographic assessment with AR and the involvement of blinded external assessors.

Residual pigmentation on Day 90 differed significantly between the two groups (*P* = .019), with severe pigmentation found exclusively in the CS group (8.3% vs 0%). However, the effect size was small (r = 0.22), statistical power for this outcome was only 36.4%, and the bootstrap CI crossed zero (see the Results section). Taken together, these features indicate that this comparison is unstable in the present sample. We therefore treat this as an exploratory secondary finding that should be interpreted with caution and should be confirmed in studies specifically powered for pigmentation, rather than as an established between-group difference.^24^

This argument is structurally supported by Bontinis et al.^7^ network meta-analysis documented a direct correlation between sclerosant potency and hyperpigmentation risk; detergents such as STS and polidocanol exhibited a consistent dose-response gradient (POL 1% > POL 0.5% > POL 0.25%), and Nd:YAG 1064 nm and HG75% ranked among the agents least likely to cause hyperpigmentation (Surface Under the Cumulative Ranking curve = 65.63 and 54.50, respectively), the two components of the LCS protocol. Rodrigues et al^25^ found convergent evidence in a within-patient RCT (n = 23), demonstrating lower pigmentation intensity in the CLaCS group by colorimetry (ΔE 1.30 vs 1.44; *P* = .027). Both studies point toward the same direction, but larger samples are needed to establish this finding robustly.

AVVQ improved significantly in both groups (*P* < .001), with no intergroup difference (*P* = .937; post hoc power = 9.1%). This was expected; AVVQ was developed for more advanced stages of venous disease and has limited discriminatory power for predominantly aesthetic complaints in CEAP C1 disease.^26^ In contrast, the patient subjective perception of aesthetic outcomes indicated a significant difference between the groups; 70.0% of patients who underwent LCS rated their result as Very Good/Excellent vs 20.9% in the CS group (*P* < .001).^5^^,^^17^^,^^27^ The CS protocol in our study was directly based on the protocol in the triple-blind RCT by Bertanha et al,^6^ which established the superiority of POL0.2% + HG70% over HG75% alone (95.2% vs 85.4%; *P* < .001), making the CS group a well-evidenced active comparator rather than a placebo. Disease-specific aesthetic scales would be more appropriate for assessing functional outcomes for future RCTs on CEAP C1.

These statistical findings should nonetheless be weighed carefully against practical considerations. Although LCS demonstrated statistically superior aesthetic outcomes across all GAIS comparisons, this benefit was obtained with substantially higher costs, equipment requirements, and procedural complexity than CS in a purely cosmetic CEAP C1 population, where the threshold for clinically meaningful benefit is inherently subjective and patient-dependent. We did not perform a formal cost-effectiveness analysis, and the transdermal laser device required for LCS is not widely available. We therefore present our findings as evidence of the statistical and mechanistic superiority of LCS rather than as a blanket recommendation for its routine adoption over CS. Future studies should formally weigh the incremental aesthetic benefit against cost and accessibility before drawing recommendations for clinical practice.

### Limitations

Our study has the 90-day follow-up as the main limitation, precluding the evaluation of venous recanalization and telangiectasia recurrence, which may occur beyond this period (reported as high at 3 years regardless of technique).^5^^,^^28^ The single-center design limits generalizability. The single-operator design ensures technical standardization but precludes executor blinding, an inherent limitation of interventional RCTs with different protocols.

Under the conservative worst-case intention-to-treat scenario, in which the two LCS noncompleters were imputed as Worse, three of the four primary GAIS comparisons (D1→D90 with AR. D30→D90 without AR, and D30→D90 with AR) remained significant after Bonferroni correction, whereas only D1→D90 without AR lost significance. Finally, we did not perform a formal cost-effectiveness analysis weighing the incremental aesthetic benefit of LCS against its additional cost and equipment requirements.

## Conclusions

Based on our findings, LCS (Nd:YAG 1064 nm + HG75%) demonstrated superiority over CS (HG67.5% + POL0.3%) for the treatment of lower limb reticular veins and telangiectasias, with superior overall aesthetic outcomes. The progressive improvement observed between Day 30 and Day 90 in the LCS group suggests an additional late therapeutic benefit of the hybrid technique. This effect may be partially explained by two complementary mechanisms: first, the synergistic vascular interaction between the transdermal laser and sclerosant, enhancing endothelial injury and delaying vessel obliteration; and second, a delayed dermal remodeling response potentially related to the photothermal interaction of Nd 1064 nm with the skin itself, a mechanism not expected in the CS group and that may contribute to continued aesthetic improvement beyond 30 days.

## Author Contributions

Conception and design: MF

Analysis and interpretation: MF, LP, RA, FG, LH, GL, EA, MR

Data collection: MF

Writing the article: MF, RA, FG, LH, MR

Critical revision of the article: MF, LP, RA, FG, LH, GL, EA, MR

Final approval of the article: MF, LP, RA, FG, LH, GL, EA, MR

Statistical analysis: MF, RA, LH, MR

Obtained funding: MF, LH, MR

Overall responsibility: MF

## Funding

This study received financial support from the Coordination for the Improvement of Higher Education Personnel (CAPES), Brazil, Finance Code 001. The Procedures were funded by Prime Vascular Clinic, and no external commercial funding was received.

## Disclosures

None.
